# Implications of hybridisation and cytotypic differentiation in speciation assessed by AFLP and plastid haplotypes - a case study of *Potentilla alpicola* La Soie

**DOI:** 10.1186/1471-2148-12-132

**Published:** 2012-08-01

**Authors:** Juraj Paule, Antonia Scherbantin, Christoph Dobeš

**Affiliations:** 1 Department of Biodiversity and Plant Systematics, Centre for Organismal Studies (COS) Heidelberg, Heidelberg University, Im Neuenheimer Feld 345, D–69120, Heidelberg, Germany; 2 Department of Botany and Molecular Evolution, Senckenberg Research Institute, Senckenberganlage 25, D–60325, Frankfurt/Main, Germany; 3 Department of Pharmacognosy, Pharmacobotany, University of Vienna, Althanstrasse 14, A–1090, Vienna, Austria

**Keywords:** AFLP, Apomixis, cpDNA, Hybridisation, Introgression, Polyploidy, Reproduction mode, *Potentilla*, Rosaceae

## Abstract

**Background:**

Hybridisation is presumed to be an important mechanism in plant speciation and a creative evolutionary force often accompanied by polyploidisation and in some cases by apomixis. The *Potentilla collina* group constitutes a particularly suitable model system to study these phenomena as it is morphologically extensively variable, exclusively polyploid and expresses apomixis. In the present study, the alpine taxon *Potentilla alpicola* has been chosen in order to study its presumed hybrid origin, identify underlying evolutionary processes and infer the discreteness or taxonomic value of hybrid forms.

**Results:**

Combined analysis of AFLP, cpDNA sequences and ploidy level variation revealed a hybrid origin of the *P. alpicola* populations from South Tyrol (Italy) resulting from crosses between *P. pusilla* and two cytotypes of *P. argentea*. Hybrids were locally sympatric with at least one of the parental forms. Three lineages of different evolutionary origin comprising two ploidy levels were identified within *P. alpicola.* The lineages differed in parentage and the complexity of the evolutionary process. A geographically wide-spread lineage thus contrasted with locally distributed lineages of different origins. Populations of *P. collina* studied in addition, have been regarded rather as recent derivatives of the hexaploid *P. argentea*. The observation of clones within both *P. alpicola* and *P. collina* suggested a possible apomictic mode of reproduction.

**Conclusions:**

Different hybridisation scenarios taking place on geographically small scales resulted in viable progeny presumably stabilised by apomixis. The case study of *P. alpicola* supports that these processes played a significant role in the creation of polymorphism in the genus *Potentilla*. However, multiple origin of hybrids and backcrossing are considered to produce a variety of evolutionary spontaneous forms existing aside of reproductively stabilised, established lineages.

## Background

Interspecific hybridisation has long been considered a potentially innovative evolutionary force playing an important role in speciation and phenotypic diversification e.g. [1-3]. Hybridisation between two (or more) distantly related species may be accompanied by doubling of the genome thus overcoming the common sterility in hybrids by providing each chromosome with a pairing partner (also referred to as allopolyploidy; [4]). Furthermore, hybridisation is also believed to be fundamental to the occurrence of apomixis (asexual reproduction through seeds), which is found almost exclusively in polyploids and highly heterozygous species [5].

Hybridisation is an important mechanism in the formation of species in the highly polymorphic genus *Potentilla*. Possible hybrid origins of several taxa associated with morphological variability, intermediacy and consequent taxonomic complexity were a concern already in the 19^th^ century (e.g. [6,7]). Later on, the presence of apomixis [8-10] and extensive intraspecific ploidy variation [11-13] supported this view and added to the understanding of the evolutionary pathways followed by the genus.

The *Potentilla collina* group from the series *Argenteae* Th. Wolf. [14] seems to be a particularly suitable model system for studying the contribution of hybridisation, polyploidisation and apomixis to the evolution of the genus. At least fifteen species [15,16] belonging to this group are considered either locally to regionally distributed microspecies and represent a taxonomically complicated Eurasian hybrid complex. The observed morphological variability and exclusive polyploidy (x = 7; 2n = 5–12x), with occasional observation of chromosome aberrations [17], are explained by the hybrid origin of the group involving taxa from the series *Aureae* Th. Wolf and *Argenteae* Th. Wolf [14,18,19]. Within the group, the development of both female and male gametophytes was reported to be disturbed or the offspring originated through apomictic pathways. Apomixis by means of apospory and pseudogamy was obligate or close to obligate [20-22]. Full or partial male sterility (9–44 %) has also been found in several studied individuals [17,21]. Furthermore, in a hexaploid *P. collina* biotype only uni- and bivalents, but no tri- or tetravalents were observed [23], which suggests the presence of at least two different genomes. Experimental hybridisations confirmed interfertility between presumed parents (e.g. [18,24]) and fertilisation of reduced (B_II_-hybrids) and unreduced egg cells (B_III_-hybrids) [24,25] have also been reported.

One example from the *P. collina* group is the *Potentilla alpicola* de La Soie, a microspecies restricted to the western and central Alps [15,26]. It occupies montane to subalpine habitats and is often found in sympatry with *P. argentea* L. and *P. pusilla* Host from the *P. verna* group [27] on population or local scales. Chromosome numbers reported so far revealed polyploidy in this taxon (2n = 5x, 6x, 12x; [26]). Concerning the morphology, *P. alpicola* is usually intermediate in most morphological characters, with some individuals tending to *P. argentea*.

The following study is based on the assumption of an autochthonous origin of *P. alpicola* and various potential parental taxa occurring in sympatry were included. We combined ploidy data with amplified fragment length polymorphisms (AFLP) and chloroplast (cp) DNA sequencing and asked four main questions: (i) Is *P. alpicola* of hybrid origin? (ii) If yes, which taxa have been involved in its formation? (iii) Did *P. alpicola* arise at several localities independently (polytopically), or did it arise in one locality and spread afterwards throughout the Central Alps? (iv) Are hybrid forms reproductively stabilised, i.e. discrete? Finally, we comment (v) on resulting taxonomic implications.

## Material and methods

### Plant material

**Figure 1:**
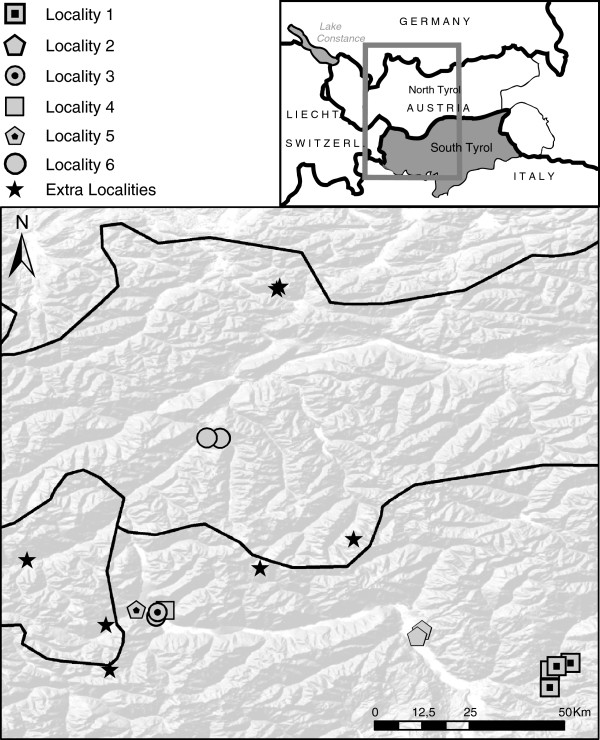
** Geographic origin of the studied populations.** A full list of localities and population codes are given in Additional file 1.

Plant material was collected from six broader localities within the central Alps (South Tyrol, Switzerland and North Tyrol) (Figure 1; Table 1; Additional file 1). *Potentilla alpicola* was sampled together with the sympatrically co-occurring possible parental taxa *P. argenteaP. pusillaP. incana* G.Gaertn., B.Mey. & Scherb]. Putative hybrid populations, morphologically deviating from *P. alpicola,* but belonging to the *P. collina* group were also sampled (referred to as “*P. collina*”). Species present in the Central Alps *P. aurea* L., *P. brauneana* Hoppe, *P. crantzii* (Crantz) Beck ex Fritsch, *P. frigida* Vill., *P. thuringiaca* Bernh. ex Link, *P. pusilla* × *thuringiaca, P.* aff. *verna* and potentially involved in the genesis of *P. alpicola* were sampled from additional six localities. Individuals were collected from a distance of at least 5 m from each other. In total, 293 accessions representing 30 populations and 11 taxa were investigated, 5–27, but mostly 10 samples per population. Herbarium vouchers from plants collected during field trips as well as from transplanted plants are deposited in HEID herbarium. In order to present the geographical data ArcGIS v9.1 (ESRI, USA) software with the Hillshade WMS-layer [28] was used.

**Table 1:** Sampling localities of studied taxa

**Locality/PopID**	**Taxon**	**Locality**
**LOC_1**		**Völs/Seis am Schlern (NE from Bozen)**
Pop088	*P. argentea*	ITA; SW of Seis am Schlern
Pop085	*P. pusilla*	ITA; Völs am Schlern, 0.5 km N
Pop089	*P. incana*	ITA; Völs am Schlern, Mongadui
Pop086	*P. alpicola*	ITA; Völs am Schlern, St. Konstantin
Pop087	*P. alpicola/pusilla*	ITA; Völs am Schlern, St. Konstantin
**LOC_2**		**Burgstall/Lana-Burgstall (SE from Meran)**
Pop093	*P. argentea*	ITA; Burgstall, western slope
Pop094	*P. pusilla*	ITA; Burgstall, western slope
Pop095	*P. collina*	ITA; Burgstall/Lana, railway station
**LOC_3**		**Glurns (Vinschgau)**
Pop098	*P. argentea*	ITA; Glurns, sedimentation tank
Pop099	*P. pusilla*	ITA; Glurns, settlement Sölles
Pop097	*P. collina*	ITA; Glurns, sedimentation tank
**LOC_4**		**Schluderns (Vinschgau)**
Pop100	*P. argentea*	ITA; Schluderns, Kalvarienberg
Pop101	*P. pusilla*	ITA; Schluderns, Kalvarienberg
Pop102	*P. alpicola*	ITA; Schluderns, Kalvarienberg
**LOC_5**		**Laatsch/Münstertal valley**
Pop198	*P. argentea*	ITA; exit of the Münstertal valley/Laatsch
Pop199	*P. pusilla*	ITA; exit of the Münstertal valley/Laatsch
Pop200	*P. alpicola*	ITA; exit of the Münstertal valley/Laatsch
**LOC_6**		**Kauns (Northern Tyrol)**
Pop206	*P. argentea*	AUT; Ötztaleralpen, Kauns, W of the church
Pop205	*P. pusilla*	AUT; Ötztaleralpen, Kauns, ESE of the church
Pop204	*P. collina*	AUT; Ötztaleralpen, Kauns, ESE of the church
**EXTRA LOCALITIES**		
Pop202	*P. argentea*	CHE; Münstertal valley, NW Müstair
Pop074	*P. aurea*	AUT; Northern Limestone Alps
Pop075	*P. brauneana*	AUT; Northern Limestone Alps
Pop080	*P. crantzii*	AUT; Obergurgl, Rotmoostal valley
Pop096	*P. frigida*	AUT; Ötztaleralpen, summit Fineiljoch
Pop103	*P. frigida*	ITA; Southern Tyrol, Stilfserjoch pass
Pop201	*P. pusilla*	CHE; Münstertal valley, NW Müstair
Pop190	*P. pusilla × thuringiaca*	CHE; Engadin, Ftan, above the railway station
Pop189	*P. thuringiaca*	CHE; Engadin, Ftan, above the railway station
Pop203	*P.* aff. *verna*	CHE; Münstertal valley, NW Müstair

Populations are ordered according to broader localities (LOC_1 – LOC_6), country codes follow ISO 3166–1 Alpha-3.

### Chromosome counts and DNA ploidy level estimation

The DNA ploidy levels were determined by flow cytometry from fresh leaf petioles using the Partec Ploidy Analyser PA (Partec, Germany) at the IPK, Gatersleben and at the Department of Pharmacognosy, University of Vienna. The samples were prepared according to the two-step (Otto) protocol [29] with the internal standard *Lycopersicon esculentum* cv. Stupické polní tyčkové rané [30]; *Potentilla incana* Ptl4311] and the nuclei were stained with 4',6-diamidino-2-phenylindole (DAPI). Sample/standard ratios were calculated from the means of the sample and standard fluorescence histograms, and only those with coefficients of variation (CVs) < 5 % for the G_0_/G_1_ peak of the analysed sample were considered. In order to obtain a reliable reference for the DNA ploidy estimation, chromosome numbers of individuals of the studied taxa were counted following Murín [31] or Dobeš [13] (see Additional file 1). In case of *P. argenteaP. incana* and *P. thuringiaca* individuals have been already karyotyped elsewhere ([32,33]). The DNA ploidy level has been attributed for each species separately based on the regression of sample/standard fluorescence ratios against the ratios of the counted individuals.

### DNA extraction, cpDNA amplification and sequencing

The total DNA was isolated from freshly-collected, silica gel-dried leaf tissue from single individuals using the procedure of Dobeš and Paule [34]. The plastid *trn*H(gug)-*psb*A intergenic spacer (IGS) was amplified using the primers: *trn*H(gug) 5’-CGC GCA TGG TGG ATT CAC AAT CC-3’ and *psb*A 5’-GTT ATG CAT GAA CGT AAT GCT C-3’ [35] and the PCR reactions were performed as described in Paule et al. [32]. The cycle sequencing was accomplished on both strands. All sequences were edited and a consensus was made of forward and reverse sequences using the software SeqMan v4.0 (DNASTAR, USA).

### AFLP analysis

The AFLP analyses were performed using the protocol established by Vos et al. [36] with few modifications as applied by Paule et al. [32]. Three differentially fluorescence labelled PCR products of the same sample were multiplexed and diluted and the fragments were separated on a MegaBase 500 DNA capillary-sequencer together with an ET-ROX 550 size standard (Amersham Biosciences, USA). In each run, a total of 48 samples were analysed, including one standard sample applied to each run, one negative control, one repeat within the runs and several other repeats (altogether 5 %). Raw data were visualised and the fragments manually scored using GeneMarker v1.8 (SoftGenetics, USA). Processed data were exported as a presence/absence matrix.

### Data analyses

The DNA-sequences were multiply aligned by means of the ClustalX v1.83 [37] and the alignments were manually refined using the GeneDoc v2.7 [38]. Two regions were excluded from the alignment due to repeated sequence motifs (poly-A stretches) and three indels were manually coded for presence and absence. Phylogenetic relationships among the cpDNA haplotypes were evaluated by means of the network analysis using the TCS v1.2 [39] with a default connection limit of 95 %.

The following statistical parameters were computed using the R-script AFLPdat ([40]; R v2.9.2 environment [41]) for the whole dataset, taxa or lineages revealed by later analyses: total number of the fragments, proportion of polymorphic fragments, number of private fragments and proportion of shared fragments among lineages. The number of different AFLP genotypes and Nei´s genotype diversity [42] in the *P. alpicola* populations were estimated using the programs Genotype v1.1 and Genodive v1.2 [43]. The functions allow entering a threshold/error rate, estimated from the observed differences among the replicates or alternatively from the observed pairwise differences between the genotypes.

In order to visualise the phylogenetic relationships among the genotypes (in a sense of AFLP phenotype as used in the following), a Neighbor-Net analysis (as implemented in SplitsTree4 v4.5; [44]) based on Jaccard distance matrix calculated beforehand with DistAFLP (accessible at http://pbil.univ-lyon1.fr/ADE-4/microb/) has been carried out. Since the relationship between hybrid taxa and their parents is not hierarchical, the similarity among AFLP genotypes was presented in a two-dimensional ordination using EUKLID [45]. EUKLID differs from alternative ordination methods in maximizing the distances among predefined groups in the mapping of data. The analysis is based on pairwise Euclidean distances. A mapping error has been calculated estimating the difference in the distance of objects in two-dimensional presentation relative to the distances of objects in the original multidimensional data matrix [46].

## Results and discussion

### DNA ploidy levels

In total, 212 individuals from 27 populations of nine studied taxa have been investigated by means of flow cytometry (see Additional file 1). Hundred and forty-one samples were measured at the IPK, Gatersleben and 71 samples at the University of Vienna [33,47]. The CVs for the G_0_/G_1_ peak of the analysed sample ranged from 1.50 to 5.13 (x[bar] = 2.70). Reference chromosome numbers were obtained for individuals of all except three taxa. The ploidy level has not been determined for *P. frigida* and *P. crantzii* populations as reference chromosome counts failed. However, based on the previously published data [48], both may be tetraploid or *P. crantzii* possibly of higher ploidy. One DNA ploidy level has been determined for *P. collina*. As no reference chromosomes were counted, the ratios were regressed with *P. argentea* counts (because of the high genetic affinity of these taxa; see later). Flow cytometric analysis also revealed that two *P. alpicola* individuals (Ptl4146, Ptl4148) could possibly be aneuhexaploid. Results for all studied taxa are summarised in Table 2.

**Table 2:** Summary of the chromosome counts and flow cytometric analyses

**Taxon**	**DNA ploidy**	***N***	**Chromosome number: individual(s) counted**	**DNA ploidy determination**
*P. alpicola*	5x	4	2n = 35: Ptl4081, Ptl4149	CC regression
	6x	36	2n = 42: Ptl4026, Ptl4141, Ptl4881,	CC regression
			Ptl4887, Ptl4911, Ptl4913	
*P. argentea*	2x	13	see Paule *et al.* 2011	CC regression
	6x	29	see Paule *et al.* 2011	CC regression
*P. aurea*	2x	5	2n = 14: Ptl3961	CC regression
*P. brauneana*	2x	3	2n = 14: Ptl3973	CC regression
*P. collina*	6x	20	–	CC regression
*P. crantzii*	possibly 4x		– (Dobeš & Vitek 2000)	Lit. review
*P. frigida*	possibly 4x		– (Dobeš & Vitek 2000)	Lit. review
*P. incana*	4x	9	see Scherbatin 2009	CC regression
*P. pusilla*	4x	26	2n = 28: Ptl4048	CC regression
	5x	11	2n = 35: Ptl4184	CC regression
	6x	1	2n = 48: Ptl4132	CC regression
	7x-	1	2n = 49: Ptl4133, Ptl4187, Ptl4188	CC count
	7x	6		CC regression
			2n = 42: Ptl4491, Ptl4497, Ptl4500	
*P. pusilla* × *P. thuringiaca*	6x	8		CC regression
			2n = 63: Ptl4571	
	9x	10		CC regression
*P. thuringiaca*			2n = 49: Ptl4325, Ptl4328	
*P.* aff. *verna*	7x	10		CC regression

CC regression: regression against counted individuals; CC count: chromosome count; Lit. review: literature review.

### AFLP analyses

**Figure 2:**
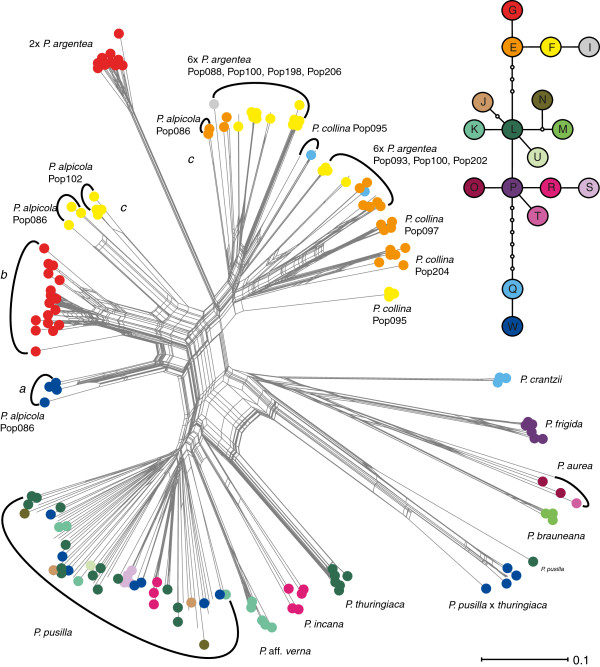
** Phylogenetic relationships inferred on the basis of AFLP data using the Neighbor-Net as implemented in SplitsTree4.** Colour-coding refers to the *trn*H-*psb*A cpDNA sequences resolved in the parsimony network depicted next to the Neighbor-Net diagram. Small empty circles represent haplotypes that are not present, but necessary to link all observed haplotypes to the network. All haplotypes are separated from the nearest haplotype by one nucleotide difference. The scale bar indicates genetic distance.

In total, 283 accessions from 30 populations were investigated; 4–27, but mostly 10 individuals per population (see Additional file 1). Three AFLP primer combinations resulted in 241 clearly scorable fragments sized from 63–537 bp and 96.68% of them were polymorphic. The number of fragments within species ranged from 58 to 205. The repeatability of the data was 98.33–100% (x[bar] =98.76%). The major splits in the Neighbor-Net separated three groups of AFLP genotypes (Figure 2): 1. *P. verna* group (including *P. thuringiaca*), 2. *P. argentea*, *P. collina*, and *P. alpicola*, 3. the *Aureae Alpestres* and the *Aureae Frigidae* including *P. pusilla × P. thuringiaca*.

### CpDNA sequence data and haplotype distribution

The cpDNA sequences were obtained for a total of 175 individuals, for at least three samples per population (see Additional file 1). The length of the *trn*H-*psb*A IGS ranged from 439 bp to 487 bp. Sixteen nucleotide substitutions, eight indels and two poly-A stretches were detected. The length of the alignment was 550 bp. After manual coding of the indels for the presence and absence and removal of the poly-A stretches, the total length of the alignment was reduced to 443 bp and 23 parsimony informative sites were considered. The sequences are deposited at NCBI GenBank (see Additional file 1).

Altogether, seventeen *trn*H-*psb*A cpDNA haplotypes were identified and the TCS network analysis revealed three groups of haplotypes (Figure 2) separated from each other by 4–12 mutations. Haplotypes E, F, G, and I representing the first group were carried by *P. argentea*[32] and the most individuals of *P. alpicola* and *P. collina* (see Additional file 1). The second group was composed of haplotypes J, K, L, M, N, O, P, R, S, T, and U and included *P. thuringiaca*, the taxa from the *P. verna* group (*P. pusillaP. incanaP.* aff. *verna*) as well as members of Wolf’s [14]*Aureae Alpestres* (*P. aurea*) and *Aureae Frigidae* (*P. brauneanaP. frigida*). Haplotypes J, K, L, N, R, S, and U were observed in individuals of AFLP genotype-group 1 only. Haplotypes M; O and T; and P were specific to *P. brauneana*; *P. aurea*; and *P. frigida*, respectively. In the third group haplotype Q was observed in hexaploid *P. argentea* (see also [32]), one individual of *P. collina* and in *P. crantzii*. Haplotype W was found in *P. pusillaP. pusilla* × *thuringiaca*, and *P. alpicola*.

### *Identity of* Potentilla alpicola *and* P. collina *individuals*

The Neighbor-Net analysis revealed different positions of *P. alpicola* in the phylogenetic network suggesting different evolutionary fates for particular populations. A majority of the individuals representing three localities (Localities 1, 4 and 5), formed a separate single cluster (Figure 2). Population Pop102 clustered with hexaploid *P. argentea,* similarly, as did the three studied populations of *P. collina*. In combination with the haplotype and cytotype data, three lineages of *P. alpicola* have been defined: pentaploids carrying haplotype W (lineage *a*), hexaploids carrying haplotype G (lineage *b*), and hexaploids grouped with hexaploid *P. argentea* carrying haplotypes E/F (lineage *c*) (Figure 2).

The three *P. alpicola* lineages possessed 115–138 AFLP fragments (Table 3). The highest proportion of fragments among all studied species was shared with *P. pusilla* (92.75–94.66 %) and hexaploid *P. argentea* (87.79–92.03 %) by all three lineages (Table 3). Similar pattern was observed for *Potentilla collina* which carried 164 fragments, 90.85 % and 87.80 % of which shared with hexaploid *P. argentea* and *P. pusilla*, respectively.

**Table 3:** AFLP fragments shared among taxa

**Nb shared fragments/ % shared fragments**	***P.alpicola*5x (a), (115)**	***P.alpicola*6x (b), (131)**	***P. alpicola*(c), (138)**	***P. collina*(164)**
*P. argentea* 2x (**78**)	**58**/50.43	**68**/51.91	**66**/47.83	**71**/43.29
*P. aurea* (**74**)	**47**/40.87	**54**/41.22	**57**/41.30	**62**/37.80
*P. brauneana* (**58**)	**44**/38.26	**47**/35.88	**45**/32.61	**49**/29.88
*P. frigida* (**66**)	**49**/42.61	**55**/41.98	**53**/38.41	**53**/32.32
*P. crantzii* (**76**)	**56/**48.70	**59**/45.04	**58**/42.03	**60**/36.59
*P. incana* (**130**)	**88**/76.52	**96**/73.28	**92**/66.67	**102**/62.20
*P. argentea* 6x (**177**)	**104**/90.43	**115**/87.79	**127**/92.03	**149**/90.85
*P. pusilla* (**205**)	**107**/93.04	**124**/94.66	**128**/92.75	**144**/87.80
*P. thuringiaca* (**156**)	**92**/80.00	**99**/75.57	**99**/71.74	**113**/68.90
*P. pusilla* × *thuringiaca* (**136**)	**87**/75.65	**87**/66.41	**88**/63.77	**101**/61.59
*P.* aff. *verna* (**118**)	**85**/73.91	**88**/67.18	**85**/61.59	**92**/56.10

The number (Nb) of fragments observed for each taxon is given in brackets right to its name. Nb of shared fragments and % of shared fragments (Nb of shared fragments/Nb of fragments in *P. alpicola* lineages, and *P. collina*, respectively) for the taxon combinations, are provided in the field of conjunction.

The following taxa had unique fragments: diploid *P. argentea* – 1 specific fragment, hexaploid *P. argentea* – 6, *P. pusilla* – 6, *P. thuringiaca* – 4, and *P. pusilla × thuringiaca* – 1. Out of these specific fragments, each *P. alpicola* lineage contained two fragments of *P. pusilla.* Additionally, *P. alpicola* lineage *c* contained one fragment of hexaploid *P. argentea. Potentilla collina* carried five specific fragments of hexaploid *P. argentea*. One specific fragment was observed for *P. collina* and *P. alpicola*. Prior to the comparisons of shared bands, individuals found in the clusters of differing taxonomy have been excluded from the analysis (*P. argentea*: Ptl4408 − 4410; *P. pusilla*: Ptl4464). These individuals were either taxonomically misidentified when collected in the field or the morphology was not reflected by the molecular data.

### Genotypic/Clonal assignment analysis

We have assumed that the same AFLP genotype represents a “clone”. If taken strictly, clones with no difference in banding patterns have been recognised in several populations of *P. alpicola* (Pop87, Pop102 and Pop200) and *P. collina* (Pop95, Pop97). However, based on the data repeatability and the pairwise differences between genotypes, a threshold of 4 and 5, respectively, has been suggested. Hence, 5 differences have been chosen as a threshold in the clonal assignment analysis (Table 4) and the analyses have been carried out for each *P. alpicola* and *P. collina* population.

**Table 4:** **Indices of clonal diversity for*P. alpicola*and*P. collina*populations**

	***Nb***	***Nb***_***geno***_	***D***_***g***_
***P. alpicola***			
Pop86	8	8	1.000
Pop87	9	3	0.417
Pop102	27	3	0.373
Pop200	9	5	0.806
***P. collina***			
Pop95	10	3	0.600
Pop97	9	2	0.500
Pop204	7	4	0.714

Measures are based on the AFLP data and computed using Genotype and Genodive. *Nb*, number of samples; *Nb*_*geno*_, number of genotypes considering a threshold of 5 fragments; *D*_*g*_, genotypic diversity.

The majority of the *P. alpicola* populations consisted of 1 or 2 abundant clones (Table 4), with an exception of a diverse population Pop86 (D_g_ = 1.00). Most of the identified clones were population specific, but one clone was shared by populations Pop102 and Pop200. Three studied *P. collina* populations were composed of 2 and 3 clones. Accordingly, we have assumed that the observed population structure can be attributed to the apomictic mode of reproduction in both *P. alpicola* and *P. collina*.

### *Evolutionary origin of* Potentilla alpicola *and* P. collina

The presence of four different chloroplast haplotypes (E, F, G, W) from two distinct haplotype groups (12 mutation steps apart; Figure 2) indicates that *P. alpicola* did not arise through a gradual differentiation but rather via other evolutionary processes. This pattern agrees with the assumed hybrid origin of *P. alpicola*. Due to the fact that the chloroplast genome is maternally inherited in the majority of angiosperms [49], the cpDNA bears on directionality of hybridisation. The three following taxa were thus identified as mothers: *P. pusilla*, diploid, and hexaploid *P. argentea*. In the case of *P. collina,* the cpDNA data suggest that only hexaploid *P. argentea* may have served as a mother. This finding is supported by the fact that *P. alpicola* as well as *P. collina* was found in close spatial proximity to these taxa.

**Figure 3:**
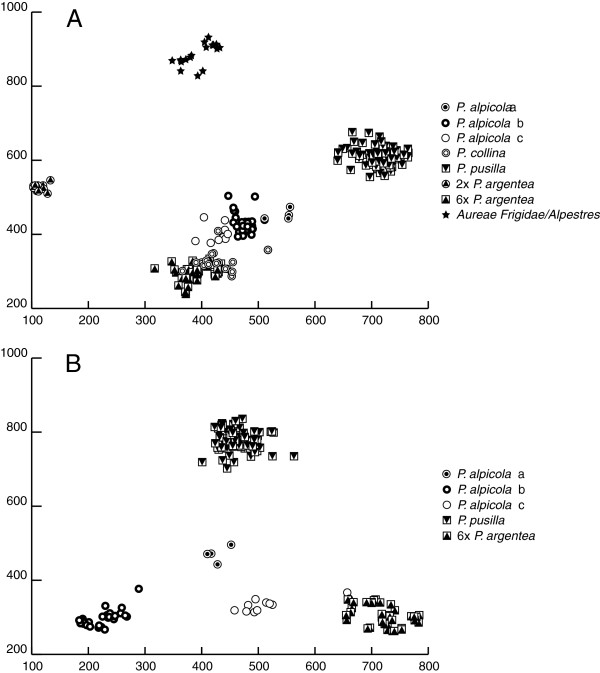
** Two-dimensional mapping of AFLP-based Euclidean distances using EUKLID. A:***Potentilla pusilla*, diploid, and hexaploid *P. argentea*, and the combined group *Aureae Frigidae (P. brauneana*, *P. frigida)/Aureae Alpestres* (*P. aurea*, *P. crantzii*) are reference groups located at the outer limits of the plot. The putative hybrids *P. alpicola* (lineages *a–c*) and *P. collina* are placed at an approximately intermediate position between *P. pusilla* and hexaploid *P. argentea*. **B:** Graphical representation of the genetic relationships of *P. alpicola* lineage *a* and lineage *c* to the reference taxa *P. pusilla*, hexaploid *P. argentea*, and *P. alpicola* lineage *b* (see text for details). The mapping error of the two-dimensional presentations is 0.187 (a) and 0.083 (b), respectively.

For further verification of this assumption, the genetic similarity of the hybrid taxa relative to these four groups was simultaneously mapped using the EUKLID ordination together with a control group *Aureae Alpestres/Aureae Frigidae*. Both, *P. collina* and *P. alpicola*, were genetically intermediate between hexaploid *P. argentea* and *P. pusilla*, but with most of the individuals closer to *P. argentea* or in case of *P. collina* partly overlapping with the *P. argentea* cluster (Figure 3a). This result supports parentage of hexaploid *P. argentea* and *P. pusilla* as a most likely scenario. In a taxonomically more focused EUKLID analysis using in addition to these two groups the *P. alpicola*-specific lineage as reference group, the remaining *P. alpicola* individuals were genetically intermediate between hexaploid *P. argentea* and the *P. alpicola*-cluster and *P. pusilla* and the *P. alpicola-*cluster, respectively (Figure 3b).

Proportions of shared AFLP fragments substantiated this finding indicated by a major nuclear contribution of hexaploid *P. argentea* and *P. pusilla* (91.72 and 89.81 % of the total shared fragments; Table 2) to the *P. alpicola* genome. The highest proportion of fragments from a diploid taxon recovered by diploid *P. argentea* also supported a contribution from this cytotype. Concerning the species-specific fragments identified in the putative parental taxa (Table 5), *P. alpicola* revealed 2 fragments from *P. pusilla*, one fragment from hexaploid *P. argentea* and no fragments from other taxa. Hence, *P. alpicola* lineages combine alleles of putative parents with an exception of one fragment, similarly as in the synthetic F1 allohexaploid between the tetraploid *Triticum turgidum* ssp. *dicocoides* and the diploid *Aegilops tauschii*[50], where the majority of the bands were additive, 17 % of both parental fragments were absent and, 2.4 % appeared de novo. The combined data thus suggest parentage of *P. pusilla* and both diploid and hexaploid *P. argentea* but with varying contributions to the *P. alpicola* genome.

**Table 5:** Summary of the molecular relationships among hybrid lineages and identified parents

**Lineage**	**Haplotype**	**Total % of AFLP bands/Taxon**	**Species-specific AFLP bands**	**Geographic origin**
*P. alpicola* 5x (a)	W	93.04/*P. pusilla*	2/6 *P. pusilla*	Pop86/Loc1
		90.43/6x *P. argentea*		
		50.43/2x *P. argentea*		
*P. alpicola* 6x (b)	G	94.66/*P. pusilla*	2/6 *P. pusilla*	Pop86/Loc1
		87.79/6x *P. argentea*		Pop87/Loc1
				Pop102/Loc4
		51.91/2x *P. argentea*		Pop200/Loc5
*P. alpicola* 6x (c)	F	92.03/*P. pusilla*	2/6 *P. pusilla*	Pop86/Loc1
		92.75/6x *P. argentea*	1/6 6x *P. argentea*	Pop87/Loc1
				Pop102/Loc4
		47.83/2x *P. argentea*		
*P. collina*	F, E	87.80/*P. pusilla*	5/6 6x *P. argentea*	Pop95/Loc2
		90.85/6x *P. argentea*		Pop97/Loc3
				Pop204/Loc6
		43.29/2x *P. argentea*		

*Potentilla collina* shared 90.85 % (149/164) of the fragments with the hexaploid *P. argentea*. Fourteen fragments were shared with *P. pusilla*. However, this is also the case in several hexaploid *P. argentea* individuals (e.g. Ptl4331–32, Ptl4335). Hence, we do not consider it an indication for a recent hybrid origin, but rather a reflection of possible introgression, which is in agreement with predominance of *P. argentea*-specific haplotypes in *P. collina*.

### Multiple versus single hybrid formation and complexity of the evolutionary process

Within our data, there is only little evidence that *P. alpicola* has a common ancestor. A majority of the studied populations possess different haplotypes and AFLP genotypes and clones were mostly population specific. The only subgroup of possibly common origin is the lineage *b* composed of individuals from three different localities (Locality 1, 4 and 5; Figure 1). The lineage possesses both a single cytotype and chloroplast haplotype and individuals from the populations Pop102 and Pop200, 8 km afar, share one AFLP genotype.

In order to verify this possibility, we asked the question, if the origin of this lineage can be explained by a single evolutionary event or if a more complex scenario should be considered. In case of a single evolutionary event, the disjunct distribution lineage *b* can be explained by dispersal. In the second case, directional selection for the observed genotypes at multiple localities has to be assumed. For that purpose, we tried to infer the ploidy of the gametes put out by the identified most likely parental species of *P. alpicola*: diploid and hexaploid *P. argentea* and *P. pusilla*. Based on the distribution of genotypic pairwise differences within the populations, a flow cytometric seed screen (Dobeš et al. unpublished research), the occurrence of anorthoploidy (whose maintenance is concomitantly coupled to asexual reproduction in *Potentilla*), and the literature record e.g. [22,51,52], regular formation of reduced egg cells via meiosis (followed by sexual fertilisation) was inferred for diploid *P. argentea* as well as tetraploid *P. pusilla*. In contrast, facultative apomeiotic origins of unreduced egg cells were found for hexaploid *P. argentea* and high polyploid (5x, 6x, 7x) *P. pusilla* cytotypes. As pollen - in contrast to female gametogenesis – in both sexual and apomictic *Potentillas* is almost exclusively produced via meiosis [20,53-55], the following likely ploidies are expected for male / female gametes: 1x / 1x diploid *P. argentea*; 3x / 6x hexaploid *P. argentea*; and 2x, 3x / 2x, 3x, 5x, 6x, and 7x *P. pusilla*.

Based on this set of parental male and female gamete ploidies, we determined possible gamete combinations resulting by fusion in the pentaploid and hexaploid *P. alpicola* genomes. Interestingly, the origin of the pentaploids (lineage *a*) only could be explained by a single crossing event as both the observed cpDNA haplotype and genetic similarities with the donating parents have met expectations. In contrast, the formation of none of the hexaploid *P. alpicola* populations (lineages *bc*) could be explained by a single event as individuals either carried a haplotype incompatible with the proposed cross and/or their genetic composition did not reflect proportions of the contributed parental genomes. This line of arguments supports the idea of a complex evolutionary history, in particular for lineage *b*. This lineage alternatively may have a single origin and subsequently dispersed to its present places of occurrence or have originated multiple times under the assumption of directional selection. Both interpretations are theoretically compatible with the recognition of these specific *P. alpicola* forms as a species. Obviously, the lineage consists of individuals characterised by a coherent combination of molecular and karyological characters. Furthermore, the origin of the pentaploid lineage *a* and the hexaploid lineage *c* may be explained by backcrosses with *P. pusilla* and *P. argentea* as suggested by intermediate position in the EUKLID (Figure 3). Hence, we consider these individuals as products of introgression of *P. alpicola* into *P. pusilla* and hexaploid *P. argentea*, respectively, or vice versa. Such rare sexual events have been documented by Holm and Ghatnekar for hexaploid apomictic *P. argentea*[51].

### Taxonomic comments

A final decision on the taxonomic status of the *P. alpicola* lineages depends on further studies of its constancy through the reproductive process and comparative autecological studies. Clonality observed within each population of *P. alpicola* (except Pop86) assumably can be attributed to the apomictic mode of reproduction as already observed in other taxa of the *P. collina* group [21,22], hexaploid *P. argentea*[51], and high polyploid *P. pusilla* (Dobeš et al. unpublished research). Autogamy associated with homozygosity may alternatively explain the pattern, but seems unlikely for the pentaploids at least as anorthoploids cytotype should not be maintained by sexuality in *Potentilla*. In any case, the observed levels of clonality suggest stable inheritance of the hybrid forms.

The lack of unique AFLP fragments and its limited geographic distribution, suggest a recent origin of *P. alpicola.* Although a coherent evolutionary lineage may be recognised among the studied *P. alpicola*-forms and accepted as a taxonomic unit following the cohesion species model [56], the widespread existence of individuals formed by backcrosses with the parents, strongly complicates the species limits. A solution to the problem may be achieved by additional efforts to complete the sampling and by the molecular approach followed here. Nevertheless, this aim is hampered by conservation issues resulting from a serious decline of populations. In Switzerland, *P. alpicola* is critically endangered and recently known from two or three localities [26], including the *locus classicus* in the Wallis [16].

## Conclusions

Combined analysis of AFLP, cpDNA sequences and ploidy levels suggested a hybrid origin of *P. alpicola* and *P. collina* populations in the South Tyrol. Diploid and hexaploid *P. argentea* and *P. pusilla* have been identified as parental taxa in different hybridisation giving rise to three lineages of *P. alpicola*. In contrast, *P. collina* populations have been regarded rather as recent derivatives of hexaploid *P. argentea*. Although the identified lineages of *P. alpicola* had different evolutionary origins, individuals from three geographically dispersed populations could possibly carry one name.

## Competing interests

The authors declare that they have no competing interests.

## Authors’ contributions

JP carried out the molecular work, flow cytometric analyses, statistical analyses, and drafted the manuscript. AS carried out flow cytometric analyses and the chromosome counting. CD designed the study, provided the samples, carried out flow cytometric analyses and drafted the manuscript. All authors read and approved the final manuscript.

## Supplementary Material

Additional file 1 **List of studied accessions and experiments.** AFLP – amplified fragment length polymorphism, FCM – flow cytometry (cc – chromosomes counted, p – presumed ploidy level based on the genetic data ^1^, - – aneuploidy). Samples are ordered according to broader localities (LOC_1 – LOC_6), country codes follows ISO 3166-1 Alpha-3. Click here for file
