# Supplementary material for: Implications of hybridisation and cytotypic differentiation in speciation assessed by AFLP and plastid haplotypes - a case study of Potentilla alpicola La Soie
Source: BMC Evol Biol. 2012 Aug 1;12:132. doi: 10.1186/1471-2148-12-132 (PMC3433326; doi:10.1186/1471-2148-12-132)
Supplement: Additional file 1 — List of studied accessions and experiments. AFLP – amplified fragment length polymorphism, FCM – flow cytometry (cc – chromosomes counted, p – presumed ploidy level based on the genetic data 1, - – aneuploidy). Samples are ordered according to broader localities (LOC_1 – LOC_6), country codes follows ISO 3166-1 Alpha-3. [file 1471-2148-12-132-S1.doc]

Additional file 1: List of studied accessions and experiments. AFLP – amplified fragment length polymorphism, FCM – flow cytometry (cc – chromosomes counted, p – presumed ploidy level based on the genetic data ^1^, - – aneuploidy). Samples are ordered according to broader localities (LOC_1 – LOC_7), country codes follows ISO 3166-1 Alpha-3.

| **Mat Nr** | **PopID** | **Taxon** | **Locality** | **AFLP** | **FCM** | **Haplotype** | **GenBank No.** | **Herbarium No.** |
| --- | --- | --- | --- | --- | --- | --- | --- | --- |
| **LOC_1**  Ptl4121  Ptl4122  Ptl4123  Ptl4124  Ptl4125  Ptl4131  Ptl4132  Ptl4133  Ptl4134  Ptl4135  Ptl4136  Ptl4137  Ptl4138  Ptl4140  Ptl4083  Ptl4201  Ptl4202  Ptl4203  Ptl4204  Ptl4205  Ptl4206  Ptl4207  Ptl4208  Ptl4209  Ptl4141  Ptl4142  Ptl4143  Ptl4145  Ptl4146  Ptl4147  Ptl4148  Ptl4149  Ptl4150  Ptl4081  Ptl4082  Ptl4151  Ptl4152  Ptl4153  Ptl4154  Ptl4155  Ptl4156  Ptl4157  Ptl4158  Ptl4159  Ptl4160  Ptl4085  Ptl4086  **LOC_2**  Ptl4171  Ptl4172  Ptl4175  Ptl4176  Ptl4177  Ptl4178  Ptl4179  Ptl4180  Ptl4181  Ptl4182  Ptl4183  Ptl4184  Ptl4185  Ptl4186  Ptl4187  Ptl4188  Ptl4189  Ptl4190  Ptl4191  Ptl4192  Ptl4193  Ptl4194  Ptl4195  Ptl4196  Ptl4197  Ptl4198  Ptl4199  Ptl4200  Ptl4089  **LOC_3**  Ptl4071  Ptl4072  Ptl4073  Ptl4074  Ptl4075  Ptl4076  Ptl4078  Ptl4079  Ptl4080  Ptl4041  Ptl4042  Ptl4043  Ptl4044  Ptl4045  Ptl4047  Ptl4048  Ptl4049  Ptl4050  Ptl4061  Ptl4063  Ptl4064  Ptl4065  Ptl4066  Ptl4067  Ptl4068  Ptl4069  Ptl4070  **LOC_4**  Ptl4401  Ptl4402  Ptl4403  Ptl4404  Ptl4405  Ptl4406  Ptl4407  Ptl4408  Ptl4409  Ptl4410  Ptl4551  Ptl4552  Ptl4553  Ptl4554  Ptl4555  Ptl4556  Ptl4557  Ptl4558  Ptl4559  Ptl4560  Ptl4888  Ptl4889  Ptl4890  Ptl4461  Ptl4462  Ptl4463  Ptl4464  Ptl4465  Ptl4466  Ptl4467  Ptl4468  Ptl4469  Ptl4470  Ptl4861  Ptl4862  Ptl4863  Ptl4864  Ptl4865  Ptl4866  Ptl4867  Ptl4868  Ptl4869  Ptl4870  Ptl4021  Ptl4022  Ptl4023  Ptl4024  Ptl4025  Ptl4026  Ptl4027  Ptl4028  Ptl4029  Ptl4030  Ptl4851  Ptl4852  Ptl4853  Ptl4854  Ptl4855  Ptl4856  Ptl4857  Ptl4858  Ptl4859  Ptl4860  Ptl4881  Ptl4882  Ptl4883  Ptl4884  Ptl4885  Ptl4886  Ptl4887  **LOC_5**  Ptl4891  Ptl4892  Ptl4893  Ptl4894  Ptl4895  Ptl4896  Ptl4897  Ptl4898  Ptl4899  Ptl4900  Ptl4901  Ptl4902  Ptl4903  Ptl4904  Ptl4905  Ptl4906  Ptl4907  Ptl4908  Ptl4909  Ptl4910  Ptl4911  Ptl4912  Ptl4913  Ptl4914  Ptl4915  Ptl4916  Ptl4917  Ptl4918  Ptl4919  **LOC_6**  Ptl4871  Ptl4872  Ptl4873  Ptl4874  Ptl4875  Ptl4876  Ptl4877  Ptl4878  Ptl4879  Ptl4880  Ptl4339  Ptl4841  Ptl4842  Ptl4843  Ptl4340  Ptl4844  Ptl4845  Ptl4846  Ptl4847  Ptl4848  Ptl4920  **EXTRA**  Ptl4331  Ptl4332  Ptl4333  Ptl4334  Ptl4335  Ptl4337  Ptl4338  Ptl3961  Ptl3963  Ptl3966  Ptl3969  Ptl3970  Ptl3971  Ptl3972  Ptl3973  Ptl3978  Ptl3979  Ptl4051  Ptl4052  Ptl4053  Ptl4054  Ptl4055  Ptl4211  Ptl4212  Ptl4213  Ptl4214  Ptl4215  Ptl4434  Ptl4435  Ptl4436  Ptl4437  Ptl4438  Ptl4571  Ptl4572  Ptl4573  Ptl4574  Ptl4575  Ptl4576  Ptl4577  Ptl4578  Ptl4579  Ptl4580  Ptl4491  Ptl4492  Ptl4493  Ptl4494  Ptl4495  Ptl4496  Ptl4497  Ptl4498  Ptl4499  Ptl4500  Ptl4341  Ptl4342  Ptl4343  Ptl4344  Ptl4345  Ptl4346  Ptl4347  Ptl4348  Ptl4321  Ptl4322  Ptl4323  Ptl4324  Ptl4325  Ptl4326  Ptl4327  Ptl4328  Ptl4329  Ptl4330 | Pop088  Pop088  Pop088  Pop088  Pop088  Pop085  Pop085  Pop085  Pop085  Pop085  Pop085  Pop085  Pop085  Pop085  Pop085  Pop089  Pop089  Pop089  Pop089  Pop089  Pop089  Pop089  Pop089  Pop089  Pop086  Pop086  Pop086  Pop086  Pop086  Pop086  Pop086  Pop086  Pop086  Pop086  Pop086  Pop087  Pop087  Pop087  Pop087  Pop087  Pop087  Pop087  Pop087  Pop087  Pop087  Pop087  Pop087  Pop093  Pop093  Pop093  Pop093  Pop093  Pop093  Pop093  Pop093  Pop094  Pop094  Pop094  Pop094  Pop094  Pop094  Pop094  Pop094  Pop094  Pop094  Pop095  Pop095  Pop095  Pop095  Pop095  Pop095  Pop095  Pop095  Pop095  Pop095  Pop095  Pop098  Pop098  Pop098  Pop098  Pop098  Pop098  Pop098  Pop098  Pop098  Pop099  Pop099  Pop099  Pop099  Pop099  Pop099  Pop099  Pop099  Pop099  Pop097  Pop097  Pop097  Pop097  Pop097  Pop097  Pop097  Pop097  Pop097  Pop100  Pop100  Pop100  Pop100  Pop100  Pop100  Pop100  Pop100  Pop100  Pop100  Pop100  Pop100  Pop100  Pop100  Pop100  Pop100  Pop100  Pop100  Pop100  Pop100  Pop100  Pop100  Pop100  Pop101  Pop101  Pop101  Pop101  Pop101  Pop101  Pop101  Pop101  Pop101  Pop101  Pop101  Pop101  Pop101  Pop101  Pop101  Pop101  Pop101  Pop101  Pop101  Pop101  Pop102  Pop102  Pop102  Pop102  Pop102  Pop102  Pop102  Pop102  Pop102  Pop102  Pop102  Pop102  Pop102  Pop102  Pop102  Pop102  Pop102  Pop102  Pop102  Pop102  Pop102  Pop102  Pop102  Pop102  Pop102  Pop102  Pop102  Pop198  Pop198  Pop198  Pop198  Pop198  Pop198  Pop198  Pop198  Pop198  Pop198  Pop199  Pop199  Pop199  Pop199  Pop199  Pop199  Pop199  Pop199  Pop199  Pop199  Pop200  Pop200  Pop200  Pop200  Pop200  Pop200  Pop200  Pop200  Pop200  Pop206  Pop206  Pop206  Pop206  Pop206  Pop206  Pop206  Pop206  Pop206  Pop206  Pop205  Pop205  Pop205  Pop205  Pop204  Pop204  Pop204  Pop204  Pop204  Pop204  Pop204  Pop202  Pop202  Pop202  Pop202  Pop202  Pop202  Pop202  Pop074  Pop074  Pop074  Pop074  Pop074  Pop075  Pop075  Pop075  Pop075  Pop075  Pop096  Pop096  Pop096  Pop096  Pop096  Pop103  Pop103  Pop103  Pop103  Pop103  Pop080  Pop080  Pop080  Pop080  Pop080  Pop189  Pop189  Pop189  Pop189  Pop189  Pop189  Pop189  Pop189  Pop189  Pop189  Pop190  Pop190  Pop190  Pop190  Pop190  Pop190  Pop190  Pop190  Pop190  Pop190  Pop201  Pop201  Pop201  Pop201  Pop201  Pop201  Pop201  Pop201  Pop203  Pop203  Pop203  Pop203  Pop203  Pop203  Pop203  Pop203  Pop203  Pop203 | *P. argentea*  *P. argentea*  *P. argentea*  *P. argentea*  *P. argentea*  *P. pusilla*  *P. pusilla*  *P. pusilla*  *P. pusilla*  *P. pusilla*  *P. pusilla*  *P. pusilla*  *P. pusilla*  *P. pusilla*  *P. pusilla*  *P. incana*  *P. incana*  *P. incana*  *P. incana*  *P. incana*  *P. incana*  *P. incana*  *P. incana*  *P. incana*  *P. alpicola*  *P. alpicola*  *P. alpicola*  *P. alpicola*  *P. alpicola*  *P. alpicola*  *P. alpicola*  *P. alpicola*  *P. alpicola*  *P. alpicola*  *P. alpicola*  *P. alpicola*  *P. alpicola*  *P. alpicola*  *P. alpicola*  *P. alpicola*  *P. alpicola*  *P. alpicola*  *P. alpicola*  *P. pusilla*  *P. alpicola*  *P. alpicola*  *P. pusilla*  *P. argentea*  *P. argentea*  *P. argentea*  *P. argentea*  *P. argentea*  *P. argentea*  *P. argentea*  *P. argentea*  *P. pusilla*  *P. pusilla*  *P. pusilla*  *P. pusilla*  *P. pusilla*  *P. pusilla*  *P. pusilla*  *P. pusilla*  *P. pusilla*  *P. pusilla*  *P. collina*  *P. collina*  *P. collina*  *P. collina*  *P. collina*  *P. collina*  *P. collina*  *P. collina*  *P. collina*  *P. collina*  *P. collina*  *P. argentea*  *P. argentea*  *P. argentea*  *P. argentea*  *P. argentea*  *P. argentea*  *P. argentea*  *P. argentea*  *P. argentea*  *P. pusilla*  *P. pusilla*  *P. pusilla*  *P. pusilla*  *P. pusilla*  *P. pusilla*  *P. pusilla*  *P. pusilla*  *P. pusilla*  *P. collina*  *P. collina*  *P. collina*  *P. collina*  *P. collina*  *P. collina*  *P. collina*  *P. collina*  *P. collina*  *P. argentea*  *P. argentea*  *P. argentea*  *P. argentea*  *P. argentea*  *P. argentea*  *P. argentea*  *P. argentea*  *P. argentea*  *P. argentea*  *P. argentea*  *P. argentea*  *P. argentea*  *P. argentea*  *P. argentea*  *P. argentea*  *P. argentea*  *P. argentea*  *P. argentea*  *P. argentea*  *P. argentea*  *P. argentea*  *P. argentea*  *P. pusilla*  *P. pusilla*  *P. pusilla*  *P. pusilla*  *P. pusilla*  *P. pusilla*  *P. pusilla*  *P. pusilla*  *P. pusilla*  *P. pusilla*  *P. pusilla*  *P. pusilla*  *P. pusilla*  *P. pusilla*  *P. pusilla*  *P. pusilla*  *P. pusilla*  *P. pusilla*  *P. pusilla*  *P. pusilla*  *P. alpicola*  *P. alpicola*  *P. alpicola*  *P. alpicola*  *P. alpicola*  *P. alpicola*  *P. alpicola*  *P. alpicola*  *P. alpicola*  *P. alpicola*  *P. alpicola*  *P. alpicola*  *P. alpicola*  *P. alpicola*  *P. alpicola*  *P. alpicola*  *P. alpicola*  *P. alpicola*  *P. alpicola*  *P. alpicola*  *P. alpicola*  *P. alpicola*  *P. alpicola*  *P. alpicola*  *P. alpicola*  *P. alpicola*  *P. alpicola*  *P. argentea*  *P. argentea*  *P. argentea*  *P. argentea*  *P. argentea*  *P. argentea*  *P. argentea*  *P. argentea*  *P. argentea*  *P. argentea*  *P. pusilla*  *P. pusilla*  *P. pusilla*  *P. pusilla*  *P. pusilla*  *P. pusilla*  *P. pusilla*  *P. pusilla*  *P. pusilla*  *P. pusilla*  *P. alpicola*  *P. alpicola*  *P. alpicola*  *P. alpicola*  *P. alpicola*  *P. alpicola*  *P. alpicola*  *P. alpicola*  *P. alpicola*  *P. argentea*  *P. argentea*  *P. argentea*  *P. argentea*  *P. argentea*  *P. argentea*  *P. argentea*  *P. argentea*  *P. argentea*  *P. argentea*  *P. pusilla*  *P. pusilla*  *P. pusilla*  *P. pusilla*  *P. collina*  *P. collina*  *P. collina*  *P. collina*  *P. collina*  *P. collina*  *P. collina*  *P. argentea*  *P. argentea*  *P. argentea*  *P. argentea*  *P. argentea*  *P. argentea*  *P. argentea*  *P. aurea*  *P. aurea*  *P. aurea*  *P. aurea*  *P. aurea*  *P. brauneana*  *P. brauneana*  *P. brauneana*  *P. brauneana*  *P. brauneana*  *P. frigida*  *P. frigida*  *P. frigida*  *P. frigida*  *P. frigida*  *P. frigida*  *P. frigida*  *P. frigida*  *P. frigida*  *P. frigida*  *P. crantzii*  *P. crantzii*  *P. crantzii*  *P. crantzii*  *P. crantzii*  *P. thuringiaca*  *P. thuringiaca*  *P. thuringiaca*  *P. thuringiaca*  *P. thuringiaca*  *P. thuringiaca*  *P. thuringiaca*  *P. thuringiaca*  *P. thuringiaca*  *P. thuringiaca*  *P. pusilla × thuringiaca*  *P. pusilla × thuringiaca*  *P. pusilla × thuringiaca*  *P. pusilla × thuringiaca*  *P. pusilla*  *P. pusilla × thuringiaca*  *P. pusilla × thuringiaca*  *P. pusilla × thuringiaca*  *P. pusilla × thuringiaca*  *P. pusilla × thuringiaca*  *P. pusilla*  *P. pusilla*  *P. pusilla*  *P. pusilla*  *P. pusilla*  *P. pusilla*  *P. pusilla*  *P. pusilla*  *P. aff. verna*  *P. aff. verna*  *P. aff. verna*  *P. aff. verna*  *P. aff. verna*  *P. aff. verna*  *P. aff. verna*  *P. aff. verna*  *P. aff. verna*  *P. aff. verna* | **Völs/Seis am Schlern (NE from Bozen)**  ITA; SW of Seis am Schlern  ITA; SW of Seis am Schlern  ITA; SW of Seis am Schlern  ITA; SW of Seis am Schlern  ITA; SW of Seis am Schlern  ITA; Völs am Schlern, 0.5 km N  ITA; Völs am Schlern, 0.5 km N  ITA; Völs am Schlern, 0.5 km N  ITA; Völs am Schlern, 0.5 km N  ITA; Völs am Schlern, 0.5 km N  ITA; Völs am Schlern, 0.5 km N  ITA; Völs am Schlern, 0.5 km N  ITA; Völs am Schlern, 0.5 km N  ITA; Völs am Schlern, 0.5 km N  ITA; Völs am Schlern, 0.5 km N  ITA; Völs am Schlern, Mongadui  ITA; Völs am Schlern, Mongadui  ITA; Völs am Schlern, Mongadui  ITA; Völs am Schlern, Mongadui  ITA; Völs am Schlern, Mongadui  ITA; Völs am Schlern, Mongadui  ITA; Völs am Schlern, Mongadui  ITA; Völs am Schlern, Mongadui  ITA; Völs am Schlern, Mongadui  ITA; Völs am Schlern, St. Konstantin  ITA; Völs am Schlern, St. Konstantin  ITA; Völs am Schlern, St. Konstantin  ITA; Völs am Schlern, St. Konstantin  ITA; Völs am Schlern, St. Konstantin  ITA; Völs am Schlern, St. Konstantin  ITA; Völs am Schlern, St. Konstantin  ITA; Völs am Schlern, St. Konstantin  ITA; Völs am Schlern, St. Konstantin  ITA; Völs am Schlern, St. Konstantin  ITA; Völs am Schlern, St. Konstantin  ITA; Völs am Schlern, St. Konstantin  ITA; Völs am Schlern, St. Konstantin  ITA; Völs am Schlern, St. Konstantin  ITA; Völs am Schlern, St. Konstantin  ITA; Völs am Schlern, St. Konstantin  ITA; Völs am Schlern, St. Konstantin  ITA; Völs am Schlern, St. Konstantin  ITA; Völs am Schlern, St. Konstantin  ITA; Völs am Schlern, St. Konstantin  ITA; Völs am Schlern, St. Konstantin  ITA; Völs am Schlern, St. Konstantin  ITA; Völs am Schlern, St. Konstantin  **Burgstall/Lana-Burgstall (SE from Meran)**  ITA; Burgstall: western slope  ITA; Burgstall: western slope  ITA; Burgstall: western slope  ITA; Burgstall: western slope  ITA; Burgstall: western slope  ITA; Burgstall: western slope  ITA; Burgstall: western slope  ITA; Burgstall: western slope  ITA; Burgstall: western slope  ITA; Burgstall: western slope  ITA; Burgstall: western slope  ITA; Burgstall: western slope  ITA; Burgstall: western slope  ITA; Burgstall: western slope  ITA; Burgstall: western slope  ITA; Burgstall: western slope  ITA; Burgstall: western slope  ITA; Burgstall: western slope  ITA; Burgstall/Lana, railway station  ITA; Burgstall/Lana, railway station  ITA; Burgstall/Lana, railway station  ITA; Burgstall/Lana, railway station  ITA; Burgstall/Lana, railway station  ITA; Burgstall/Lana, railway station  ITA; Burgstall/Lana, railway station  ITA; Burgstall/Lana, railway station  ITA; Burgstall/Lana, railway station  ITA; Burgstall/Lana, railway station  ITA; Burgstall/Lana, railway station  **Glurns (Vinschgau)**  ITA; Glurns: sedimentation tank  ITA; Glurns: sedimentation tank  ITA; Glurns: sedimentation tank  ITA; Glurns: sedimentation tank  ITA; Glurns: sedimentation tank  ITA; Glurns: sedimentation tank  ITA; Glurns: sedimentation tank  ITA; Glurns: sedimentation tank  ITA; Glurns: sedimentation tank  ITA; Glurns – settlement Sölles  ITA; Glurns – settlement Sölles  ITA; Glurns – settlement Sölles  ITA; Glurns – settlement Sölles  ITA; Glurns – settlement Sölles  ITA; Glurns – settlement Sölles  ITA; Glurns – settlement Sölles  ITA; Glurns – settlement Sölles  ITA; Glurns – settlement Sölles  ITA; Glurns: sedimentation tank  ITA; Glurns: sedimentation tank  ITA; Glurns: sedimentation tank  ITA; Glurns: sedimentation tank  ITA; Glurns: sedimentation tank  ITA; Glurns: sedimentation tank  ITA; Glurns: sedimentation tank  ITA; Glurns: sedimentation tank  ITA; Glurns: sedimentation tank  **Schluderns (Vinschgau)**  ITA; Schluderns, Kalvarienberg  ITA; Schluderns, Kalvarienberg  ITA; Schluderns, Kalvarienberg  ITA; Schluderns, Kalvarienberg  ITA; Schluderns, Kalvarienberg  ITA; Schluderns, Kalvarienberg  ITA; Schluderns, Kalvarienberg  ITA; Schluderns, Kalvarienberg  ITA; Schluderns, Kalvarienberg  ITA; Schluderns, Kalvarienberg  ITA; Schluderns, Kalvarienberg  ITA; Schluderns, Kalvarienberg  ITA; Schluderns, Kalvarienberg  ITA; Schluderns, Kalvarienberg  ITA; Schluderns, Kalvarienberg  ITA; Schluderns, Kalvarienberg  ITA; Schluderns, Kalvarienberg  ITA; Schluderns, Kalvarienberg  ITA; Schluderns, Kalvarienberg  ITA; Schluderns, Kalvarienberg  ITA; Schluderns, Kalvarienberg  ITA; Schluderns, Kalvarienberg  ITA; Schluderns, Kalvarienberg  ITA; Schluderns, Kalvarienberg  ITA; Schluderns, Kalvarienberg  ITA; Schluderns, Kalvarienberg  ITA; Schluderns, Kalvarienberg  ITA; Schluderns, Kalvarienberg  ITA; Schluderns, Kalvarienberg  ITA; Schluderns, Kalvarienberg  ITA; Schluderns, Kalvarienberg  ITA; Schluderns, Kalvarienberg  ITA; Schluderns, Kalvarienberg  ITA; Schluderns, Kalvarienberg  ITA; Schluderns, Kalvarienberg  ITA; Schluderns, Kalvarienberg  ITA; Schluderns, Kalvarienberg  ITA; Schluderns, Kalvarienberg  ITA; Schluderns, Kalvarienberg  ITA; Schluderns, Kalvarienberg  ITA; Schluderns, Kalvarienberg  ITA; Schluderns, Kalvarienberg  ITA; Schluderns, Kalvarienberg  ITA; Schluderns, Kalvarienberg  ITA; Schluderns, Kalvarienberg  ITA; Schluderns, Kalvarienberg  ITA; Schluderns, Kalvarienberg  ITA; Schluderns, Kalvarienberg  ITA; Schluderns, Kalvarienberg  ITA; Schluderns, Kalvarienberg  ITA; Schluderns, Kalvarienberg  ITA; Schluderns, Kalvarienberg  ITA; Schluderns, Kalvarienberg  ITA; Schluderns, Kalvarienberg  ITA; Schluderns, Kalvarienberg  ITA; Schluderns, Kalvarienberg  ITA; Schluderns, Kalvarienberg  ITA; Schluderns, Kalvarienberg  ITA; Schluderns, Kalvarienberg  ITA; Schluderns, Kalvarienberg  ITA; Schluderns, Kalvarienberg  ITA; Schluderns, Kalvarienberg  ITA; Schluderns, Kalvarienberg  ITA; Schluderns, Kalvarienberg  ITA; Schluderns, Kalvarienberg  ITA; Schluderns, Kalvarienberg  ITA; Schluderns, Kalvarienberg  ITA; Schluderns, Kalvarienberg  ITA; Schluderns, Kalvarienberg  ITA; Schluderns, Kalvarienberg  **Laatsch/Münstertal valley**  ITA; exit of the Münstertal valley/Laatsch  ITA; exit of the Münstertal valley/Laatsch  ITA; exit of the Münstertal valley/Laatsch  ITA; exit of the Münstertal valley/Laatsch  ITA; exit of the Münstertal valley/Laatsch  ITA; exit of the Münstertal valley/Laatsch  ITA; exit of the Münstertal valley/Laatsch  ITA; exit of the Münstertal valley/Laatsch  ITA; exit of the Münstertal valley/Laatsch  ITA; exit of the Münstertal valley/Laatsch  ITA; exit of the Münstertal valley/Laatsch  ITA; exit of the Münstertal valley/Laatsch  ITA; exit of the Münstertal valley/Laatsch  ITA; exit of the Münstertal valley/Laatsch  ITA; exit of the Münstertal valley/Laatsch  ITA; exit of the Münstertal valley/Laatsch  ITA; exit of the Münstertal valley/Laatsch  ITA; exit of the Münstertal valley/Laatsch  ITA; exit of the Münstertal valley/Laatsch  ITA; exit of the Münstertal valley/Laatsch  ITA; exit of the Münstertal valley/Laatsch  ITA; exit of the Münstertal valley/Laatsch  ITA; exit of the Münstertal valley/Laatsch  ITA; exit of the Münstertal valley/Laatsch  ITA; exit of the Münstertal valley/Laatsch  ITA; exit of the Münstertal valley/Laatsch  ITA; exit of the Münstertal valley/Laatsch  ITA; exit of the Münstertal valley/Laatsch  ITA; exit of the Münstertal valley/Laatsch  **Kauns (Northern Tyrol)**  AUT; Ötztaleralpen, Kauns, W of the church  AUT; Ötztaleralpen, Kauns, W of the church  AUT; Ötztaleralpen, Kauns, W of the church  AUT; Ötztaleralpen, Kauns, W of the church  AUT; Ötztaleralpen, Kauns, W of the church  AUT; Ötztaleralpen, Kauns, W of the church  AUT; Ötztaleralpen, Kauns, W of the church  AUT; Ötztaleralpen, Kauns, W of the church  AUT; Ötztaleralpen, Kauns, W of the church  AUT; Ötztaleralpen, Kauns, W of the church  AUT; Ötztaleralpen, Kauns, ESE of the church  AUT; Ötztaleralpen, Kauns, ESE of the church  AUT; Ötztaleralpen, Kauns, ESE of the church  AUT; Ötztaleralpen, Kauns, ESE of the church  AUT; Ötztaleralpen, Kauns, ESE of the church  AUT; Ötztaleralpen, Kauns, ESE of the church  AUT; Ötztaleralpen, Kauns, ESE of the church  AUT; Ötztaleralpen, Kauns, ESE of the church  AUT; Ötztaleralpen, Kauns, ESE of the church  AUT; Ötztaleralpen, Kauns, ESE of the church  AUT; Ötztaleralpen, Kauns, ESE of the church  CHE; Münstertal valley, NW Müstair  CHE; Münstertal valley, NW Müstair  CHE; Münstertal valley, NW Müstair  CHE; Münstertal valley, NW Müstair  CHE; Münstertal valley, NW Müstair  CHE; Münstertal valley, NW Müstair  CHE; Münstertal valley, NW Müstair  AUT; Northern Limestone Alps  AUT; Northern Limestone Alps  AUT; Northern Limestone Alps  AUT; Northern Limestone Alps  AUT; Northern Limestone Alps  AUT; Northern Limestone Alps  AUT; Northern Limestone Alps  AUT; Northern Limestone Alps  AUT; Northern Limestone Alps  AUT; Northern Limestone Alps  AUT; Ötztaleralpen, summit Fineiljoch  AUT; Ötztaleralpen, summit Fineiljoch  AUT; Ötztaleralpen, summit Fineiljoch  AUT; Ötztaleralpen, summit Fineiljoch  AUT; Ötztaleralpen, summit Fineiljoch  ITA; Southern Tyrol, Stilfserjoch pass  ITA; Southern Tyrol, Stilfserjoch pass  ITA; Southern Tyrol, Stilfserjoch pass  ITA; Southern Tyrol, Stilfserjoch pass  ITA; Southern Tyrol, Stilfserjoch pass  AUT; Obergurgl, Rotmoostal valley  AUT; Obergurgl, Rotmoostal valley  AUT; Obergurgl, Rotmoostal valley  AUT; Obergurgl, Rotmoostal valley  AUT; Obergurgl, Rotmoostal valley  CHE; Engadin, Ftan, above the railway station  CHE; Engadin, Ftan, above the railway station  CHE; Engadin, Ftan, above the railway station  CHE; Engadin, Ftan, above the railway station  CHE; Engadin, Ftan, above the railway station  CHE; Engadin, Ftan, above the railway station  CHE; Engadin, Ftan, above the railway station  CHE; Engadin, Ftan, above the railway station  CHE; Engadin, Ftan, above the railway station  CHE; Engadin, Ftan, above the railway station  CHE; Engadin, Ftan, above the railway station  CHE; Engadin, Ftan, above the railway station  CHE; Engadin, Ftan, above the railway station  CHE; Engadin, Ftan, above the railway station  CHE; Engadin, Ftan, above the railway station  CHE; Engadin, Ftan, above the railway station  CHE; Engadin, Ftan, above the railway station  CHE; Engadin, Ftan, above the railway station  CHE; Engadin, Ftan, above the railway station  CHE; Engadin, Ftan, above the railway station  CHE; Münstertal valley, NW Müstair  CHE; Münstertal valley, NW Müstair  CHE; Münstertal valley, NW Müstair  CHE; Münstertal valley, NW Müstair  CHE; Münstertal valley, NW Müstair  CHE; Münstertal valley, NW Müstair  CHE; Münstertal valley, NW Müstair  CHE; Münstertal valley, NW Müstair  CHE; Münstertal valley, NW Müstair  CHE; Münstertal valley, NW Müstair  CHE; Münstertal valley, NW Müstair  CHE; Münstertal valley, NW Müstair  CHE; Münstertal valley, NW Müstair  CHE; Münstertal valley, NW Müstair  CHE; Münstertal valley, NW Müstair  CHE; Münstertal valley, NW Müstair  CHE; Münstertal valley, NW Müstair  CHE; Münstertal valley, NW Müstair | X  X  X  X  X  X  X  X  X  X  X  X  X  X  X  X  X  X  X  X  X  X  X  X  X  X  X  X  X  X  X  X  X  X  X  X  X  X  X  X  X  X  X  X  X  X  X  X  X  X  X  X  X  X  X  X  X  X  X  X  X  X  X  X  X  X  X  X  X  X  X  X  X  X  X  X  X  X  X  X  X  X  X  X  X  X  X  X  X  X  X  X  X  X  X  X  X  X  X  X  X  X  X  X  X  X  X  X  X  X  X  X  X  X  X  X  X  X  X  X  X  X  X  X  X  X  X  X  X  X  X  X  X  X  X  X  X  X  X  X  X  X  X  X  X  X  X  X  X  X  X  X  X  X  X  X  X  X  X  X  X  X  X  X  X  X  X  X  X  X  X  X  X  X  X  X  X  X  X  X  X  X  X  X  X  X  X  X  X  X  X  X  X  X  X  X  X  X  X  X  X  X  X  X  X  X  X  X  X  X  X  X  X  X  X  X  X  X  X  X  X  X  X  X  X  X  X  X  X  X  X  X  X  X  X  X  X  X  X  X  X  X  X  X  X  X  X  X  X  X  X  X  X  X  X  X  X  X  X  X  X  X  X  X  X  X  X  X  X  X  X  X  X  X  X  X  X  X  X  X  X  X  X | 6x  2x  2x  6x  2x  7x  cc_7x  cc_7x  5x  5x  7x  5x  5x  5x  7x  4x  4x  4x  4x  4x  4x  4x  4x  4x  cc_6x  5x  5x  6x-  6x-  cc_5x  6x  cc_5x  6x  cc_6x  6x  6x  6x  6x  6x  6x  6x  6x  6x  6x  6x(-)  6x  6x  6x  6x  6x  6x  6x  6x  5x  5x  5x  cc_5x  5x  5x  cc_7x  cc_7x  5x  5x  6x  6x  6x  6x  6x  6x  6x  6x  6x  6x  6x  2x  2x  2x  2x  2x  2x  2x  2x  2x  4x  4x  4x  4x  4x  cc_4x  4x  4x  6x  6x  6x  6x  6x  6x  6x  6x  6x  6x  p2x  p6x  p6x  p2x  p2x  p6x  p6x  p6x  p6x  p2x  p6x  p6x  p6x  p2x  p2x  p6x  p2x  p6x  p2x  6x  p2x  p6x  4x  4x  4x  6x  4x  4x  4x  4x  4x  4x  4x  4x  4x  4x  4x  4x  6x  6x  6x  cc_6x  6x  6x  6x  cc_6x  6x  6x  6x  6x  cc_6x  6x  6x  6x  6x  6x  6x  6x  6x  6x  6x  4x  4x  4x  4x  4x  6x  4x  4x  4x  4x  cc_6x  6x  cc_6x  6x  6x  6x  6x  6x  6x  p6x  p6x  p6x  p6x  p6x  p6x  p6x  p6x  p6x  6x  6x  6x  6x  6x  6x  6x  2x  6x  cc_2x  2x  2x  2x  2x  cc_2x  2x  2x  9x  9x  9x  9x  9x  9x  9x  9x  9x  9x  cc_6x  6x  6x  6x  4x  cc_6x  6x  6x  cc_6x  4x  4x  4x  4x  4x  4x  4x  4x  7x  7x  7x  7x  cc_7x  7x  7x  cc_7x  7x  7x | F  G  G  S  S  S  L  L  R  R  R  R  R  G  G  W  W  E  F  E  W  F  W  E  G  G  G  G  F  S  F  F  F  F  F  R  R  R  L  L  F  F  F  F  F  Q  G  G  G  G  G  W  L  J  W  W  E  E  E  E  E  E  G  G  F  G  E  E  I  E  E  G  N  L  L  G  K  K  L  K  J  N  F  F  F  F  G  G  G  G  G  G  G  G  G  F  F  F  F  E  W  W  L  W  L  G  G  G  G  G  G  G  G  G  F  F  F  F  F  F  W  W  W  W  E  E  E  E  E  E  E  Q  F  E  E  G  O  T  O  M  M  M  P  P  P  P  P  P  Q  Q  Q  L  L  L  L  L  L  L  L  W  W  L  W  W  L  L  U  K  L  K  K  K  K  K  K | HM776564  HM776524  GQ384983  HQ433319  HQ433279  HQ433324  HQ433326  HQ433268  HQ433267  HQ433266  HQ433327  HQ433323  HQ433263  HQ433280  HQ433281  HQ433282  HQ433264  HQ433320  HM776563  HQ433318  HQ433278  HM776523  HQ433322  HQ433265  HM776542  HM776527  HM776529  HM776543  HM776568  HM776545  HM776530  HQ433317  HQ433275  HQ433276  HQ433277  HQ433283  HQ433284  HQ433285  HM776556  HM776546  HQ433325  HQ433286  HQ433287  HQ433288  HQ433289  HQ433290  HQ433291  HM776567  HM776555  HQ433331  HQ433274  HQ433271  HQ433272  HQ433269  HQ433270  HM776573  HM776526  GQ384978  GQ384979  GQ384981  HQ433321  HQ433330  HQ433299  HQ433300  HQ433301  HQ433302  HQ433303  HQ433304  HQ433305  HQ433306  HQ433328  HQ433298  HQ433297  HQ433296  HQ433295  HQ433294  HQ433293  HQ433292 | HEID 807094, - 807095  HEID 807096, - 807097  HEID 807098, - 807099  HEID 807100, - 807101  HEID 807102, - 807103  HEID 807112, - 807113  HEID 807114, - 807115  HEID 807116, - 807117  HEID 807118, - 807119  HEID 806886, - 807120  HEID 807121, - 807122  HEID 807123, - 807124  HEID 807125, - 807126  HEID 807128  HEID 807030  HEID 807266  HEID 807267  HEID 807268, - 807269  HEID 807270, - 807271  HEID 807272, - 807273, - 807274  HEID 807275, - 807276  HEID 807277, - 807278  HEID 807279, - 807280  HEID 807281, - 807282  HEID 807130, - 807131, - 807132  HEID 807133, - 807134  HEID 807135, - 807136  HEID 807138, - 807139  HEID 807140, - 807141  HEID 807142, - 807143  HEID 807144, - 807145  HEID 807146, - 807147  HEID 807148, - 807149  HEID 807025, - 807026  HEID 807027, - 807028, - 807029  HEID 807150, - 807151  HEID 807152, - 807153  HEID 807154, - 807155  HEID 807156, - 807157, - 807158, - 807159  HEID 807160, - 807161, - 807162, - 807163  HEID 807164, - 807165  HEID 807166, - 807167, - 807168  HEID 807169, - 807170, - 807171  HEID 807172, - 807173  HEID 807174, - 807175  HEID 807031  HEID 807032, - 807033  HEID 807193, - 807194  HEID 807195, - 807196, - 807197  HEID 807200, - 807201  HEID 807202, - 807203  HEID 807205, - 807206, - 807207  HEID 807208, - 807209, - 807210  HEID 807211, - 807212, - 807213  HEID 807214, - 807215, - 807216  HEID 807217, - 807218  HEID 807219, - 807220  HEID 807221, - 807222  HEID 807223, - 807224, - 807225  HEID 807226, - 807227, - 807228  HEID 807229, - 807230  HEID 807231, - 807232, - 807233  HEID 807234, - 807235  HEID 807236, - 807237, - 807238  HEID 807239, - 807240  HEID 807241, - 807242  HEID 807243, - 807244, - 807245  HEID 807246, - 807247  HEID 807248, - 807249, - 807250  HEID 807251, - 807252  HEID 807253, - 807254  HEID 807255, - 807256, - 807257, - 807258  HEID 807259, - 807260  HEID 807261, - 807262  HEID 807263, - 807264, - 807265  HEID 807040, - 807041  HEID 807007, - 807008  HEID 807009, - 807010  HEID 807011, - 807012  HEID 807013, - 807014  HEID 807015, - 807016  HEID 807017, - 807018  HEID 807020, - 807021  HEID 807022, - 807023  HEID 807024  HEID 806959, - 806960  HEID 806961, - 806962  HEID 806963, - 806964  HEID 806965, - 806966  HEID 806967, - 806968  HEID 806970, - 806971  HEID 806972, - 806973  HEID 806974, - 806975  HEID 806976  HEID 806987, - 806988  HEID 806990, - 806991  HEID 806992, - 806993  HEID 806994, - 806995  HEID 806996, - 806997  HEID 806998, - 806999  HEID 807000, - 807001, - 807002  HEID 807003, - 807004  HEID 807005, - 807006  HEID 807545, - 807546, - 807547, 807548  HEID 807698  HEID 807699  HEID 807700  HEID 807555  HEID 806883  HEID 806923, - 806924, - 806925  HEID 806926, - 806927  HEID 806928, - 806929, - 806930  HEID 806931  HEID 806932, 806933  HEID 806934  HEID 806935, - 806936, - 806937  HEID 806938  HEID 806939, - 806940, - 806941  HEID 807667  HEID 807668  HEID 807669  HEID 807670  HEID 807671  HEID 807672  HEID 807673  HEID 807674  HEID 807675  HEID 807676  HEID 807688, - 807689  HEID 807690  HEID 807691  HEID 807692  HEID 807693, - 807694  HEID 807695, - 807696  HEID 807697  HEID 807701, 807702  HEID 807703, 807704  HEID 807705, 807706  HEID 807707, 807708  HEID 807709, 807710  HEID 807711, 807712  HEID 807713, 807714  HEID 807715, 807716, 807717  HEID 807718  HEID 807719, 807720  HEID 807721  HEID 807722  HEID 807723  HEID 807724  HEID 807725  HEID 807726  HEID 807727  HEID 807728  HEID 807729  HEID 807730  HEID 807731, 807732  HEID 807733, 807734  HEID 807735  HEID 807736, 807737  HEID 807738  HEID 807739, 807740  HEID 807741, 807742  HEID 807743, 807744  HEID 807745, 807746, 807747  HEID 807677  HEID 807678  HEID 807679  HEID 807680  HEID 807681  HEID 807682  HEID 807683  HEID 807684  HEID 807685  HEID 807686, 807687  HEID 807475  HEID 807658  HEID 807659  HEID 807660  HEID 807476  HEID 807661  HEID 807662  HEID 807663  HEID 807664  HEID 807665  HEID 807748  HEID 807457, 807458, 807459  HEID 807460, 807461  HEID 807462, 807463  HEID 807464, 807465  HEID 807466, 807467, 807468  HEID 807471, 807472  HEID 807473, 807474  HEID 805691  HEID 805693, - 805694  HEID 805697, - 805698  HEID 805701, - 805702  HEID 805703  HEID 805704  HEID 805705  HEID 805712  HEID 806884, - 806977  HEID 806978  HEID 806979  HEID 806980, - 807813  HEID 806981  HEID 807284  HEID 807285  HEID 807286  HEID 807287  HEID 807288            HEID 807592  HEID 807593  HEID 807594  HEID 807595  HEID 807596  HEID 807597  HEID 807598  HEID 807599  HEID 807600  HEID 807601  HEID 807570  HEID 807571  HEID 807572  HEID 807573  HEID 807574  HEID 807575  HEID 807576  HEID 807577  HEID 807578  HEID 807579  HEID 807477  HEID 807478  HEID 807479  HEID 807480  HEID 807481  HEID 807482  HEID 807483  HEID 807484  HEID 807447  HEID 807448  HEID 807449  HEID 807450  HEID 807451  HEID 807452  HEID 807453  HEID 807454  HEID 807455  HEID 807456 |

^1^ Paule J, Sharbel TF & Dobeš C 2011. Apomictic and sexual lineages of the Potentilla argentea L. group (Rosaceae) – cytotype and molecular genetic differentiation. Taxon 60: 721-732.
